# The N2 During Preschool: Temporal Stability and a Test of Bidirectional Effects With Maternal Emotion Characteristics in a White, European‐American Sample

**DOI:** 10.1111/cdev.70004

**Published:** 2025-07-18

**Authors:** Anahid Akbaryan, Reese C. Burkey, Peter J. Ramirez, Ashley L. Walker, JungWon Choi, Sejal Mistry‐Patel, Jennifer L. Kling, Rebecca J. Brooker

**Affiliations:** ^1^ Department of Psychological and Brain Sciences Texas A&M University College Station Texas USA

**Keywords:** inhibitory control, maternal emotion, N2, preschool

## Abstract

Despite well‐documented behavioral changes, the development of neuropsychological substrates underlying inhibitory control remains unknown, hindering understanding of this construct over time. Stability and change in N2, a neural correlate of inhibitory control, and its crosslagged, bidirectional associations with maternal emotion characteristics were examined in 121 preschoolers (59% female, predominantly White) between 2014 and 2017. N2 was stable from 3 to 5 years of age. Greater maternal negativity at age 4 predicted smaller N2 amplitudes in preschoolers at age 5 (*β* = 0.22); preschoolers' N2 amplitudes did not predict subsequent emotion characteristics in mothers. Findings provide initial evidence for mother‐to‐child, but not child‐to‐mother effects linking N2 with mothers' emotion characteristics and a foundation for future longitudinal work on developing neural correlates of inhibitory control in preschoolers.

Inhibitory control, an early‐emerging component of child temperament, refers to children's ability to self‐regulate behaviors through the volitional suppression of prepotent actions (Derryberry and Rothbart 1997). Inhibitory control is a fundamental component of broader executive functioning, along with working memory and cognitive control; together these skills are considered essential for healthy cognitive development and later achievement among young children (Diamond 2013). Conversely, impairments in inhibitory control are linked to heightened risk for a range of psychological disorders (Berger and Buttelmann 2022).

Behavioral work suggests that during preschool, inhibitory control shows marked gains, as children transition from mostly reflexive and reactive behaviors to increasingly intentional responses to environmental signals (Derryberry and Rothbart 1997; Geeraerts et al. 2021). Though behavioral changes are well documented, the development of the neuropsychological substrates that underlie inhibitory control remains uncharacterized. This gap in knowledge is important given that a major challenge for charting the development of inhibitory control has been age‐related heterotypic continuity in measurable behaviors (Petersen et al. 2016). Moreover, a sole focus on behavioral observations precludes an understanding of whether apparent influences on inhibitory control, such as maternal emotion characteristics, are impactful at the level of function for processes that support observed behavior. In this study, we investigated stability and change in a neural marker of inhibitory control during preschool and its bidirectional associations with maternal emotion characteristics.

## 1 Early Childhood Development of Inhibitory Control

Early behavioral studies offer evidence that inhibitory control abilities increase rapidly over the preschool years (Wiebe et al. 2012). The most pronounced improvements are observed in early childhood. For example, studies of children between 3 and 5.25 years of age suggest increasingly strategic response inhibition abilities by 3.75 years (Wiebe et al. 2012). Parent‐reported data also indicate inhibitory control increases between ages 2.5 and 6.5, though the rate of growth decelerates even as abilities continue to improve (Geeraerts et al. 2021).

Developmentally sensitive neuroscientific approaches allow for the examination of neural markers of inhibitory control in young children. These studies generally use a child‐friendly version of a go/no‐go task (Hoyniak 2017) that requires a button‐press response to frequently presented “go” stimuli and response inhibition to infrequently presented “no‐go” stimuli. The N2 (Hoyniak 2017), an event‐related potential (ERP) believed to index inhibitory control, can be isolated from time‐locked electroencephalogram (EEG) recordings. The N2 appears as the second negative deflection in EEG recordings, approximately 200–400 ms after the presentation of a stimulus. In general, larger N2 amplitudes in response to no‐go trials, relative to go trials, are believed to indicate greater inhibitory control (Hosch et al. 2024) though better inhibitory control has also been linked to smaller no‐go N2 amplitudes (Espinet et al. 2012; Hoyniak and Petersen 2019) and other work reports null associations (Hoyniak and Petersen 2019). One possible explanation for contrasting patterns of links between no‐go N2 and response inhibition may be linked to task complexity, believed to be positively associated with N2 amplitudes (Espinet et al. 2012), or to normative changes in N2 over time that preclude a stable association (Hoyniak 2017; Hoyniak and Petersen 2019); at least one study controlling for age links larger N2 to better inhibition. The possibility that changing associations between no‐go N2 and function may be linked to typical development underscores the importance of delineating a typical developmental trajectory of N2 over time.

Inhibitory control indexed by the N2 likely develops in early childhood in ways that are consistent with observed inhibitory control behaviors (Best and Miller 2010). Mean‐level N2 amplitudes appear to decrease across childhood, with older children showing less negative amplitudes than younger children (Hoyniak 2017), though these patterns, too, are inconsistent across childhood (Cragg et al. 2009). Decreases in N2 amplitudes are interpreted as increases in inhibitory control efficiency and the normative consequence of increasing skull thickness as children grow (Lewis et al. 2006).

Although previous studies provide valuable preliminary insights into developing mechanisms of inhibitory control, this literature remains limited for at least three reasons. First, nearly all existing work on early childhood N2 has been cross‐sectional. In some instances, longitudinal work is under way (Hosch et al. 2024), but is not yet complete enough to analyze longitudinal trajectories. As such, conclusions about development have been inferred from comparisons across age groups or individual studies. Such an approach has been important for calling attention to the putative role of the N2 in early inhibitory control development, but is insufficient for a true understanding of developmental change. Second, many prior developmental studies have used age groups that are both broadly defined and well beyond the preschool period. Because neural development is rapid during early childhood, broad age ranges can result in conclusions about early development that are driven by the presence of the oldest children in the sample (Hoyniak 2017). As such, an overrepresentation of older children in existing work may be obscuring a clear understanding of the development of inhibitory control at the neural level during one of its most critical periods of development. To address these existing weaknesses, we investigated longitudinal changes in the N2 during the preschool years (ages 3–5), with assessments intentionally structured to minimize variability in participant age at each assessment.

Finally, in addition to being conducted with older children, the majority of earlier studies on age‐related changes in N2 have focused only on absolute stability, limiting our understanding of relative stability. Absolute or mean‐level stability refers to changes or similarities in the total level or amount of a specific behavior over time (Forehand and Jones 2002). For example, if the average inhibitory control score of a group of children remains the same over a period, it demonstrates absolute stability. In contrast, relative or rank‐order stability refers to the constancy of an individual's rank order within a group over time for some measure or variable (Forehand and Jones 2002). This would, for example, involve assessing whether children who have high inhibitory control relative to their peers at one time point still rank high at a later time point. Relative stability is viewed as critical for investigations of individual differences that can offer insight into associations between N2 and individual‐level predictors of change. Therefore, in this study we approached the investigation of stability and change of N2 using both absolute and relative approaches.

## 2 Child Inhibitory Control and Maternal Emotion Characteristics

Though originally viewed as fixed, temperament traits are accepted as at least partially malleable over time (Shiner et al. 2012). Per the Bioecological Model (Bronfenbrenner and Morris 2007), development occurs through reciprocal interactions between children and their environment, particularly with caregivers. Likely because they comprise the majority of primary caregivers in the United States (Herbst 2023), mothers are particularly salient influences on young children's development, including the acquisition of self‐regulatory skills such as inhibitory control (Kopp 1982).

Mothers can influence the development of inhibitory control in offspring in a variety of ways, many of which operate through their own emotional characteristics. Such processes are outlined in both *scaffolding* and *imitation* models of development within parent–child interactions. Rooted in classic developmental theory extolling the benefits of infants' high‐quality interactions with competent social partners (Vygotsky 1978), scaffolding theory spotlights intentional parental guidance in children's acquisition of inhibitory control (Hughes and Ensor 2009). Parents may intentionally structure children's environments in ways that allow for intermediate, increasing successes at self‐regulation. As a child develops, less scaffolding is needed from parents, and independent regulation is achieved.

In contrast, imitation models highlight unintentional consequences of children's observations of parent behavior (Dunn 1993), whereby children may observe and then demonstrate parents' emotion behaviors. Consistent with both perspectives, parents who are better able to regulate displays of their own negative emotions (Shaffer and Obradović 2017) are more likely to employ the types of parenting behaviors that directly support the development of self‐regulation and inhibitory control in children (Valiente et al. 2007).

On the other hand, behaviors that undermine the development of self‐regulatory skills in children are linked to high levels of negative maternal emotion. Maternal negativity, representing a global level of negative emotions, includes frequent expressions of sadness, fear, hostility, and fatigue (Watson and Clark 1994). Greater negative emotions in mothers predict less regulatory ability, including inhibitory control, in children (Eisenberg et al. 2001). When parents express high levels of negative emotion toward their children, children may—consistent with an imitation model—come to internalize parents' behaviors, ultimately hindering the development of regulation (Eisenberg et al. 2001). Such a process is likely also compounding over time, as parents' negative emotion expressions lead to overarousal in their children, further undermining developing self‐regulatory skills (Hoffman 1983).

It is not entirely clear in the extant literature whether the general level of maternal negativity is itself critical for child development or whether measures of general negativity may proxy heightened negative emotionality in more specific domains. Such a distinction may be important for increasing specificity in developmental models of risk and early programs of prevention. Maternal anxiety, in particular, is negatively associated with numerous domains of cognitive control in children (Goodman et al. 2016). This negative relation might be explained by a tendency for mothers with more anxiety symptoms to use nonsupportive reactions or fail to respond sensitively to their children's negative emotions (Breaux et al. 2016). Maternal anxiety, in particular, is linked to the same behaviors that theoretical models posit will interfere with the development of inhibitory control in young children. Specifically, maternal anxiety symptoms are linked to propensities for overcontrol and appear to lead to overstructured and less autonomous environments for offspring (Jones et al. 2021) impairing opportunities to practice skills like inhibitory control under structured guidance (Kopp 1982). The scaffolding model would make a clear prediction for such overcontrolling tendencies to predict impaired long‐term development of children's inhibitory control. Indeed, heightened maternal anxiety in preschool predicts lower levels of inhibitory control in early childhood (Geeraerts et al. 2021).

A limited body of work has identified relations between maternal emotion and child inhibitory control at the level of neural processing, as indexed by the N2. Greater maternal engagement and support toward children predicts greater N2 amplitudes in preschoolers (Moilanen et al. 2010) and mothers who are more emotionally supportive may model effective inhibitory control behaviors and strategies (Swingler et al. 2018). Nonetheless, little work has attempted to parse apart the role of broad (i.e., negativity) from domain‐specific (i.e., anxiety symptoms) maternal emotion characteristics as risk factors that directly impact children's N2. Understanding potential differences between broad and domain‐specific processes and their role in children's developing N2 can help to more precisely define pathways of risk and inform prevention and intervention efforts aimed at increasing inhibitory control in early childhood. As such, an additional aim of this work is to understand if and when maternal negativity and maternal anxiety independently predict the development of N2 during preschool.

Finally, it is important to note that even classic models identify critical roles for reciprocal, or bidirectional, effects in the mother–child dyad (Bronfenbrenner and Morris 2007). Thus, although most previous research has focused on the impact of maternal emotional characteristics on children's inhibitory control, it is also possible that a child's inhibitory control can influence the mother's emotional characteristics. Children with poor inhibitory control may challenge their parent's capacity to stay sensitive and refrain from intrusive behavior, ultimately increasing parent levels of negativity (Morasch and Bell 2011). Indeed, there is evidence that child characteristics can impact maternal anxiety symptoms over time (Brooker et al. 2015, 2023), with more negative behaviors in children predicting greater levels of parent anxiety over time. Similarly, work with older children has shown that intervention‐related reductions in anxiety symptoms are associated with contemporaneous reductions in parent symptoms even if parents are uninvolved in the intervention (Silverman et al. 2009). Yet, to our knowledge, no work has directly investigated putative links between preschoolers' developing inhibitory control at the neural level and parent emotion characteristics, an effort that could greatly advance our understanding of the role of early inhibitory control in the early mother–child dyad. Thus, our final goal was to test this possibility, modeling crosslagged bidirectional effects between maternal emotional characteristics and preschoolers' N2.

## 3 The Current Study

The first objective of the current study was to conduct a confirmatory test of longitudinal changes in preschoolers' inhibitory control as indexed by the N2 component from age 3 to 5 years. Based on cross‐sectional work, we hypothesized that N2 amplitudes would decrease over time. The second objective was to conduct a confirmatory test of the bidirectional relations between child N2 and maternal emotion characteristics. We hypothesized bidirectional associations such that greater negative emotion in mothers would predict less inhibitory control (more positive N2 amplitudes) in preschoolers over time. However, given evidence for associations between maternal anxiety and overcontrol, we hypothesized that more anxiety symptoms in mothers would predict less inhibitory control (less negative N2 amplitudes) in preschoolers over time. We also hypothesized that greater inhibitory control (more negative N2 amplitudes) would predict less negativity and fewer anxiety symptoms in mothers at subsequent assessments. This is the first study to explore these relations longitudinally, offering possible insight into the bidirectional effects of child neural development and maternal emotional characteristics.

## 1 Method

### 1.1 Participants

Data were collected between 2014 and 2017 as part of a larger, longitudinal study of socioemotional development across the preschool years (Brooker, 2018; Mistry‐Patel & Brooker, 2023). Data collection for the parent project was approved by the Institutional Review Board at Montana State University (Title: Convergent Markers of Risk for Psychopathology in Young Children; Number: RB070213‐FC) and analyses for the current work were approved by Institutional Review Board at Texas A&M University (Title: Biological and Environmental Influences on Emotional Development in Young Children; Number: IRB2020‐0099D). Families were recruited from the greater Bozeman, MT area through advertisements, fliers posted in local business and doctors’ offices, direct mailings based on published birth records, in‐person recruitment events, and word‐of‐mouth. Inclusion criteria required that children be typically developing, not taking any stimulant medications, and free of any known neurological impairments (self and first‐degree relatives). In total, 121 preschoolers were enrolled (59% female).

Preschoolers visited the laboratory with their primary caregiver three times during the study: ages 3.5 years (*n* = 108; *M*_age_ = 3.59, SD = 0.15), 4.5 years (*n* = 98; *M*_age_ = 4.57, SD = 0.15), and 5.5 years (*n* = 91; *M*_age_ = 5.52, SD = 0.12). At the initial visit, mothers' ages ranged from 19.75 to 45.84 years (*M*_age_ = 34.53). Rolling recruitment procedures resulted in variable sample sizes across the three assessments, though retention for the sample was excellent (90.7% retained from 3.5 to 4.5 years and 92.9% retained from 4.5 to 5.5 years).

Most mothers (95.6%) and fathers (94.8%) identified as White, 1.8% of mothers and 2.1% of fathers identified as Asian, and 1.8% of mothers and 3.8% of fathers identified as American Indian or Alaska Native. About 2% (1.8%) of mothers and 4.3% of fathers identified as Hispanic or Latino.

Of those parents who reported annual income, 1.7% reported earning less than $15,000, 4.3% reported $15,001–$20,000, 7.0% reported $20,001–$30,000, 5.2% reported $30,001–$40,000, 10.4% reported $40,001–$50,000, 17.4% reported $50,001–$60,000, 9.6% reported $60,001–$70,000, 8.7% reported $70,001–$80,000, 9.6% reported $80,001–$90,000, and 26.1% reported earning $90,001 or greater.

### 1.2 Procedure

At each age, children and their primary caregiver completed a laboratory assessment that included the recording of physiological data during computerized tasks and observations of child behaviors during standardized emotion‐elicitation paradigms. Parents (primary and secondary caregiver, when applicable) completed packets of questionnaires approximately 1 week prior to the laboratory visit that included questions about family demographics, child temperament, and parent mental health. Given study hypotheses, this report focuses on EEG data collected during a modified go/no‐go paradigm along with mothers' self‐reports of negative emotion and anxiety symptoms.

### 1.3 Measures

#### 1.3.1 Inhibitory Control N2

At each laboratory visit, children independently completed a modified go/no‐go task designed to assess aspects of cognitive control and self‐regulation (Brooker 2018). Before starting the task, an experimenter provided instructions to the child using laminated pictures of either a cartoon spaceship or an asteroid. The experimenter instructed the child to press a response button placed on a table in front of them when presented with an asteroid (the go stimulus) and to “wait” when presented with a spaceship (no‐go stimulus). After demonstrating an understanding of the task using the laminated pictures (see Supporting Information for details), children completed two practice blocks of the task, each consisting of 10 trials and using the same pictures, on a computer. Following the practice blocks, children completed two experimental blocks, each comprising 40 trials, for a maximum total of 80 trials per participant. Trials were identical to practice trials. Computerized trial‐to‐trial feedback was provided during practice trials to facilitate learning the task; all participants were rewarded with a sticker and verbal encouragement (“you're doing great”) at the end of each practice and experimental block.

Each trial began with the presentation of a fixation cross for 200 ms in the center of a 23‐in. computer monitor using Presentation stimulus delivery software (Neurobehavioral Systems, Berkeley, CA). Following this, stimuli were presented for a duration of 1200 ms. The fixation cross was then presented again for 300–800 ms before the next trial. Trials were pseudorandomized to ensure that roughly 60% of trials were go‐trials. In an effort to equate task difficulty across participants, similar to other work (Brooker 2018; Lewis et al. 2006), stimulus presentation times decreased by 50 ms following two consecutive correct responses; stimulus presentation time increased by 50 ms following two consecutive incorrect responses.

Continuous EEG was recorded using BioSemi's Active 2 recording system (Cortech Solutions, Wilmington, NC). Ag‐AgCl‐tipped electrodes were arranged in a 64‐channel cap according to the standard 10–20 labeling system. Data were sampled at a rate of 2048 Hz and referenced to the Common Mode Sense and Driven Right Leg electrodes during recording. Passive electrodes were placed at the outer canthi of the left and right eye to aid in the identification of eye blinks and movements during data processing. Passive electrodes were also placed on each wrist to obtain electrocardiogram data (not used here) and on each mastoid for possible offline rereferencing, though data were ultimately rereferenced to the whole head average because it resulted in less data loss.

**FIGURE 1:**
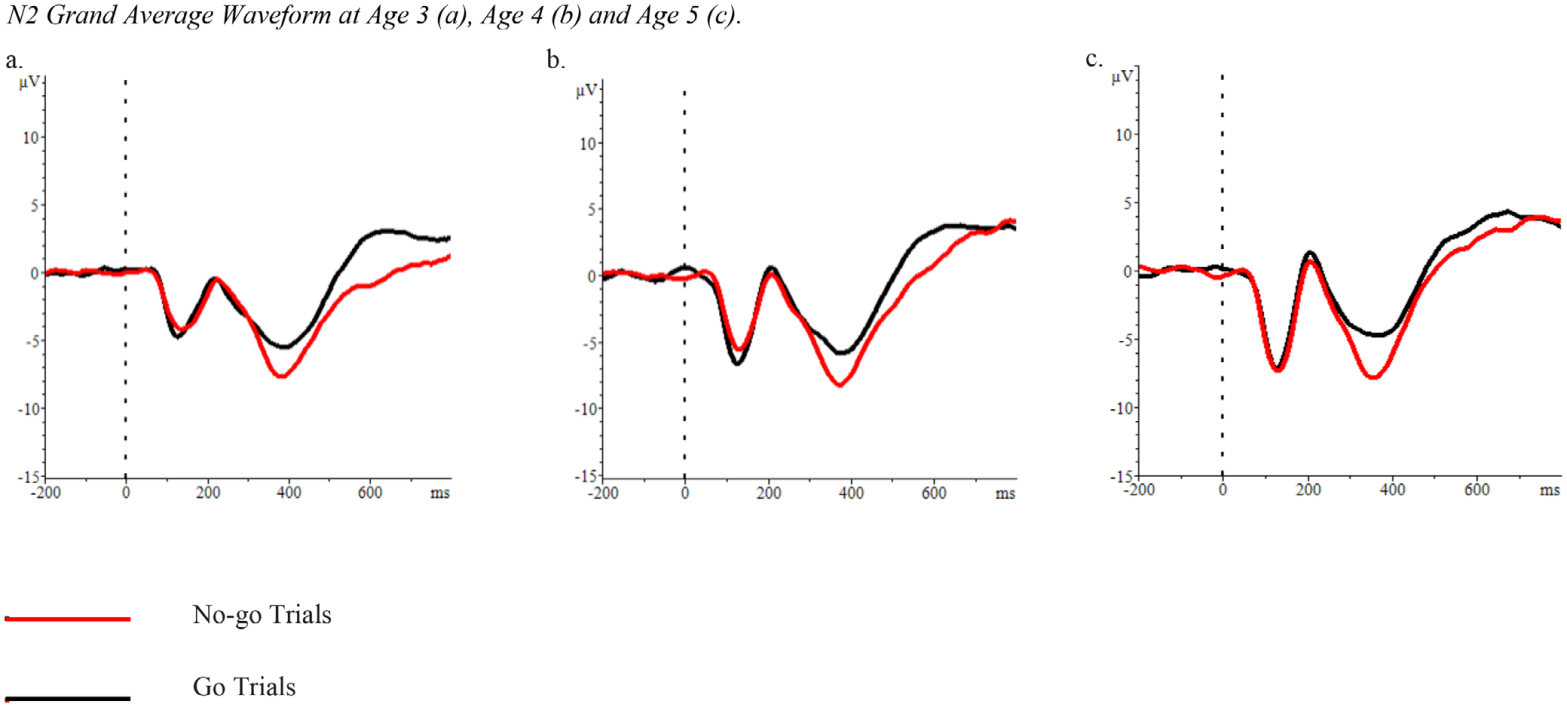
N2 grand average waveform at age 3 (a), age 4 (b), and age 5 (c).

Offline, bad channels were interpolated and electrodes were rereferenced to the whole head average, high‐pass filtered at 0.1 Hz and low‐pass filtered at 20 Hz. Go and No‐go trials were segmented (−200 to 800 ms) and baseline corrected for 200 ms prior to the response. Artifacts were identified using an automated procedure when one of the following criteria was met: a difference of 150 μV within 200 ms, a voltage step of more than 75 μV between data points, amplitudes below 0.05 μV within a 50 ms period, or activity that exceeded +100 or −100 μV. Artifact‐free trials were averaged within conditions (go and no‐go) for each participant. Peak amplitudes were identified between 200 and 500 ms and mean amplitudes at the peak (±40 ms; Luck 2014) were exported at electrode Cz. The time window and electrode site were identified based on previous work (Hoyniak 2017) and, because our sample is younger than those typically represented in the literature, confirmed through an examination of the grand average waveforms (Figure 1). Amplitudes in the no‐go condition were regressed onto amplitudes in the go condition to isolate activity specific to trials requiring inhibitory control. Individual residual scores were saved for each participant as an operationalization of N2.

#### 1.3.2 Maternal Negativity

General negativity in mothers was assessed using the Positive and Negative Affect Schedule—Expanded Form (PANAS‐X; Watson and Clark 1994). The PANAS‐X is a 60‐item inventory that asks respondents to rate, on a 5‐point Likert scale (1 = *very slightly or not at all*, 5 = *extremely*), the degree to which they generally feel different emotions or feelings (e.g., irritable, sluggish, joyful, etc.). Although the instrument allows for variations in the directions to indicate the time frame (e.g., feelings in the past few weeks), no time frame was indicated in order to encourage the report of “typical” feelings. The PANAS‐X produces five scales that reflect negativity: fatigue (4 items), guilt (6 items), sadness (6 items), fear (7 items), and hostility (6 items). The instrument also offers a negative affect composite that is partially redundant with items from the lower order scales (10 items). Because we did not have a priori hypotheses about which domains of maternal negativity would be most relevant to children's cognitive control, we used the lower order scales for our latent variable analysis in order to allow for the retention of the most coherent domains of negativity for mothers in our sample. Internal consistency was acceptable at each age across all scales (age 3 [fatigue *α* = 0.85, guilt *α* = 0.90, sadness *α* = 0.87, fear *α* = 0.90, hostility *α* = 0.83], age 4 [fatigue *α* = 0.86, guilt *α* = 0.94, sadness *α* = 0.91, fear *α* = 0.91, hostility *α* = 0.61], age 5 [fatigue *α* = 0.84, guilt *α* = 0.92, sadness *α* = 0.89, fear *α* = 0.90, hostility *α* = 0.83]).

We conducted a preliminary principal components analysis (PCA) in order to identify a plausible data‐driven single composite of maternal negativity from the PANAS‐X that could be confirmed in the measurement model. At each age, the scale reflecting feelings of guilt had less variability relative to other scales, resulting in a low communality. As such, the PCA that fit best (communalities and factor loadings > 0.40 for all items) and accounted for the greatest proportion of variance in the original scales at each age was a composite of the four remaining scales (age 3: average communality = 1.00, mean factor loading = 1.00, 99.89% of total variance; age 4: average communality = 0.60, mean factor loading = 0.75, 60.00% of total variance; age 5: average communality = 0.70, mean factor loading = 0.83). Thus, these scales were retained for the latent variable of maternal negativity.

#### 1.3.3 Maternal Anxiety Symptoms

Symptoms of anxiety in mothers were assessed as a latent composite of self‐reported anxiety symptoms across three questionnaires intended to capture different dimensions of anxiety: the Generalized Anxiety Disorder Questionnaire (GADQ‐IV; Newman et al. 2002), the Social Interaction Anxiety Scale (SIAS; Mattick and Clarke 1998), and the Penn State Worry Questionnaire (Meyer et al. 1990).

##### 1.3.3.1 GADQ‐IV

The GADQ‐IV is a 14‐item inventory that assesses symptoms of worry consistent with DSM‐5 criteria for Generalized Anxiety Disorder. Mothers provided ratings of yes or no (e.g., *do you experience excessive worry*) regarding their experiences of worry. Mothers who reported high levels of worry in the last 6 months were also asked to rate, on a 9‐point Likert scale, the degree to which symptoms were impairing (e.g., *how much do worry and physical symptoms interfere with your life, work, social activities, family; 0 = no distress, 8 = very severely*).

Consistent with standardized scoring procedures, if a mother responded “no” to item 6, administration was discontinued, allowing for a maximum of 7 points for the first six items, which assess core symptoms of GAD including excessive and uncontrollable worry. Additional points indicated associated impairment for those who already met GAD criteria on the initial six items. Total scores range from 0 to 12 with scores of greater than 5.7 indicating a likely presence of Generalized Anxiety Disorder (Newman et al. 2002). Internal consistency was acceptable at each phase (age 3: *α* = 0.93, age 4: *α* = 0.76, age 5: *α* = 0.86). A subset of mothers exceeded the clinical cutoff (> 5.7 points) for GAD at each age (age 3: 18.56%, age 4: 2.27%, age 5: 20.78%). Changes in maternal anxiety symptoms appeared not to be due to selective attrition (see Supporting Information).

##### 1.3.3.2 PSWQ

The PSWQ is a 16‐item survey that measures worry, a key feature of Generalized Anxiety Disorder (Meyer et al. 1990). Mothers indicated, using a 5‐point Likert scale, the degree to which statements regarding the occurrence and pervasiveness of worry (e.g., *I am always worrying about something*) were characteristic of them (1 = *not at all typical*, 5 = *very typical*). Ratings for each item were summed, resulting in a total possible score of 80. Internal consistency was acceptable at each phase (age 3: *α* = 0.95, age 4: *α* = 0.95, age 5: *α* = 0.95). Scores greater than 62 indicate a possible presence of Generalized Anxiety in unselected samples (Behar et al. 2003). A subset of mothers exceeded this cutoff at each age (age 3: 11.46%, age 4: 10.47%, age 5: 10.39%).

##### 1.3.3.3 SIAS

The SIAS is a 20‐item questionnaire that measures maternal symptoms consistent with a diagnosis of Social Phobia (e.g., *when mixing socially, I am uncomfortable*). Mothers rated, using a 5‐point Likert scale, the degree to which statements were characteristic of them (0 = *not at all*, 4 = *extremely characteristic*). Items correspond to the Diagnostic and Statistical Manual, Third Edition—Revised (DSM‐III) and are similar to diagnostic criteria in the DSM‐5, with the exception that the DSM‐5 expanded criteria to include social situations not exclusive to a performance setting. Responses were summed across items, resulting in a total possible score of 80. Internal consistency was high (age 3: *α* = 0.94, age 4: *α* = 0.94, age 5: *α* = 0.93). A subset of mothers exceeded the clinical cutoff (> 36 points) for likely Social Phobia (Peters 2000) at each phase (age 3: 18.75%, age 4: 23.86%, age 5: 19.74%).

### 1.4 Missing Data

Of those who provided EEG recordings, 6 children did not have usable N2s at age 3, 12 children did not have usable N2s at age 4, and 9 children did not have usable N2s at age 5.

Nine mothers were missing questionnaire data at age 3; 4 mothers completed the questionnaire packet but selectively skipped the PSWQ; 3 mothers selectively skipped the SIAS. Ten mothers were missing questionnaire data at age 4.5. Fourteen mothers were missing questionnaire data at age 5.5. Missing data were largely a result of excessive artifact (N2) or mothers not returning questionnaires, though additional data were missing at age 5.5 due to the PI changing institutions, resulting in an end to the study before all visits could be completed.

An analysis of patterns of missing data (Little 1988) suggested that data were missing at random (Little's MCAR *χ* ^2^(556) = 592.00, *p* = 0.141). As such, missing data were handled during data analysis using a Full‐Information Maximum Likelihood procedure that leverages all available data, allowing one to take advantage of the full sample from which at least some data are available, and producing unbiased parameter estimates (Enders 2010).

### 1.5 Plan for Analysis

Correlations and descriptive statistics were examined for individual variables prior to beginning the main analysis. Following this, a measurement model was tested to confirm the structure of latent maternal negativity and anxiety variables. The measurement model contained only a test of the factor structure and included no parameters reflecting associations between variables. Fit of the measurement model was assessed against several common indices and published recommendations for evaluation. Specifically, model fit was considered adequate if the root mean square error of approximation (RMSEA; Browne and Cudeck 1993), Comparative Fit Index (CFI; Bentler 1990), and Tucker‐Lewis Index (TLI; Tucker and Lewis 1973) met previously established guidelines (Browne and Cudeck 1993).

Once the measurement model was confirmed, we built the structural model to test primary hypotheses. Stability and change over time were tested by modeling autoregressive pathways for assessments of child N2, maternal negativity, and maternal anxiety between each assessment. Bidirectional effects comprised both mother‐to‐child and child‐to‐mother crosslagged pathways. Mother‐to‐child effects were tested as pathways from maternal anxiety and maternal negativity at child ages 3.5 and 4.5 to child N2 at ages 4.5 and 5.5, respectively. Child‐to‐mother effects were tested as pathways from child N2 at ages 3.5 and 4.5 to maternal anxiety and negativity at child ages 4.5 and 5.5, respectively.

## 2 Results

### 2.1 Descriptive Statistics, First‐Order Associations, and Mean‐Level Changes Among Variables

As indicated in Table 1, all self‐report ratings of maternal emotion appeared largely stable over time.

**TABLE 1:** Descriptive statistics for raw variables.

	Minimum	Maximum	Mean	SD
**Maternal anxiety symptoms**				
GADQ age 3	0.00	12.00	2.82	3.54
GADQ age 4	0.00	10.42	1.43	1.83
GADQ age 5	0.00	10.58	2.74	3.21
PSWQ age 3	18.00	75.00	43.76	14.36
PSWQ age 4	18.00	70.00	42.36	14.47
PSWQ age 5	16.00	73.00	43.29	13.75
SIAS age 3	3.00	70.00	26.09	13.61
SIAS age 4	5.00	63.00	26.81	13.74
SIAS age 5	3.00	59.00	25.93	12.37
**Maternal negativity**				
Fatigue age 3	5.00	20.00	12.79	3.67
Fatigue age 4	4.00	20.00	12.38	3.90
Fatigue age 5	4.00	19.00	11.28	3.78
Sad age 3	6.00	29.00	12.97	4.92
Sad age 4	6.00	27.00	13.66	5.85
Sad age 5	4.00	25.00	12.55	5.73
Fear age 3	7.00	35.00	13.80	5.59
Fear age 4	7.00	28.00	14.14	5.73
Fear age 5	3.00	33.00	12.76	5.19
Hostile age 3	6.00	24.00	12.92	4.55
Hostile age 4	6.00	25.00	12.71	4.38
Hostile age 5	5.00	26.00	12.49	4.21
**Child cognitive control**				
N2 age 3	−14.81	−2.80	−9.69	2.16
N2 age 4	−14.03	1.37	−4.51	2.52
N2 age 5	−9.59	0.29	−3.89	1.90

*Note:* Each measure is on a different scale and so separate measures cannot be directly compared. *Sad* and *hostile* refer to scales of sadness and hostility, respectively, but are abbreviated to accommodate table dimensions.

Consistent with the waveforms depicted in Figure 1, mean N2 amplitudes appeared to decrease slightly over time.

Although some changes in mean amplitudes may reflect increasing variability over time, particularly between ages 3 and 4, a decrease in amplitudes over time is consistent with expectations based on changes in skull thickness. A repeated measures ANOVA confirmed a significant decrease in amplitude over time (*F*(2, 90) = 167.42, *p* < 0.01), comprising a significant decline in N2 from ages 3 to 4 (*M*_diff_ = 5.37, *p* < 0.01) but a nonsignificant decrease between ages 4 and 5 (*M*_diff_ = 0.64, *p* = 0.24), suggesting a possible slowing of the rate of change over time.

Correlations among raw variables (Table 2) underscored consistency in all measures over time. Reports of maternal anxiety were also largely correlated across measures, supporting the latent variable approach. Maternal anxiety and maternal negativity were partially, though not wholly overlapping, supporting their inclusion as separate variables. For the most part, self‐report measures were more highly correlated than observed N2, reflecting both expectations for developmental change and shared method variance.

**TABLE 2:** Bivariate correlations among raw variables.

	1.	2.	3.	4.	5.	6.	7.	8.	9.	10	11.	12.	13.	14.	15.	16.	17.	18.	19.	20.	21.	22.	23.
1. GADQ age 3																							
2. GADQ age 4	**0.45**																						
3. GADQ age 5	**0.55**	**0.51**																					
4. PSWQ age 3	**0.79**	**0.40**	**0.52**																				
5. PSWQ age 4	0.**57**	**0.63**	**0.64**	**0.68**																			
6. PSWQ age 5	**0.55**	**0.47**	**0.78**	**0.72**	**0.80**																		
7. SIAS age 3	**0.51**	**0.35**	0.20	**0.53**	**0.43**	*0.25*																	
8. SIAS age 4	**0.42**	**0.44**	**0.35**	**0.54**	**0.56**	**0.37**	**0.78**																
9. SIAS age 5	**0.37**	**0.41**	**0.46**	**0.45**	**0.58**	**0.53**	**0.61**	**0.80**															
10. Fatigue age 3	**0.36**	0.14	0.19	**0.34**	*0.30*	**0.35**	**0.32**	**0.35**	**0.31**														
11. Fatigue age 4	0.15	*0.22*	0.18	0.10	*0.26*	0.16	0.09	*0.24*	0.17	**0.43**													
12. Fatigue age 5	*0.26*	0.14	**0.34**	0.22	*0.25*	**0.38**	0.14	*0.29*	**0.47**	**0.49**	**0.60**												
13. Sad age 3	**0.47**	0.28	**0.34**	**0.39**	**0.43**	**0.52**	**0.36**	**0.45**	**0.46**	**0.62**	**0.38**	**0.35**											
14. Sad age 4	*0.29*	0.37	**0.36**	**0.34**	**0.49**	**0.48**	*0.26*	**0.38**	**0.39**	0.21	**0.59**	**0.33**	**0.52**										
15. Sad age 5	0.23	0.21	**0.39**	0.19	*0.30*	**0.48**	0.11	0.24	**0.34**	0.21	**0.41**	**0.47**	**0.46**	**0.51**									
16. Fear age 3	**0.51**	**0.38**	**0.37**	**0.46**	**0.54**	0.**47**	**0.31**	**0.49**	**0.41**	**0.60**	**0.30**	*0.28*	**0.76**	**0.36**	0.21								
17. Fear age 4	*0.27*	**0.39**	**0.45**	**0.32**	**0.56**	**0.50**	0.07	**0.30**	*0.27*	0.19	**0.54**	*0.28*	**0.37**	**0.75**	**0.47**	**0.42**							
18. Fear age 5	*0.28*	0.15	**0.45**	*0.27*	**0.44**	**0.54**	0.00	0.21	**0.30**	0.19	*0.30*	**0.52**	*0.27*	**0.36**	**0.68**	*0.25*	**0.66**						
19. Hostile age 3	**0.43**	*0.25*	**0.35**	**0.38**	**0.47**	**0.48**	0.19	**0.38**	**0.38**	**0.55**	**0.37**	*0.25*	0.**73**	**0.36**	0.24	**0.73**	**0.48**	*0.31*					
20. Hostile age 4	*0.26*	**0.28**	0.20	*0.25*	**0.36**	*0.28*	0.15	0.21	0.16	*0.23*	**0.63**	*0.30*	**0.36**	**0.64**	**0.33**	**0.34**	**0.66**	**0.33**	**0.50**				
21. Hostile age 5	0.21	0.14	**0.36**	0.17	*0.25*	**0.39**	0.06	0.11	0.18	0.15	**0.44**	**0.45**	*0.27*	**0.34**	**0.71**	0.10	0.44	**0.66**	**0.33**	**0.50**			
22. N2 age 3	0.08	−0.04	−0.02	−0.03	−0.08	−0.15	0.08	0.04	−0.03	−0.04	−0.09	−0.00	−0.17	−0.03	−0.06	−0.13	0.00	0.01	−0.11	−0.08	0.09		
23. N2 age 4	0.11	0.10	0.06	0.08	0.00	0.02	0.03	0.01	−0.08	0.11	*0.25*	0.04	−0.01	0.04	0.03	−0.03	0.09	0.11	0.07	*0.24*	*0.33*	0.**39**	
24. N2 age 5	0.03	0.07	0.05	0.02	0.14	0.08	0.02	−0.05	−0.08	0.18	0.23	−0.01	−0.02	0.17	0.05	−0.02	0.22	−0.05	−0.03	*0.28*	0.08	*0.27*	**0.46**

*Note:* Italics indicate correlations that are significant at a threshold of *p* < 0.05, bold indicates correlations that are significant at a threshold of *p* < 0.01. *Sad* and *hostile* refer to scales of sadness and hostility, respectively, but are abbreviated to accommodate table dimensions.

### 2.2 Measurement Model

**FIGURE 2:**
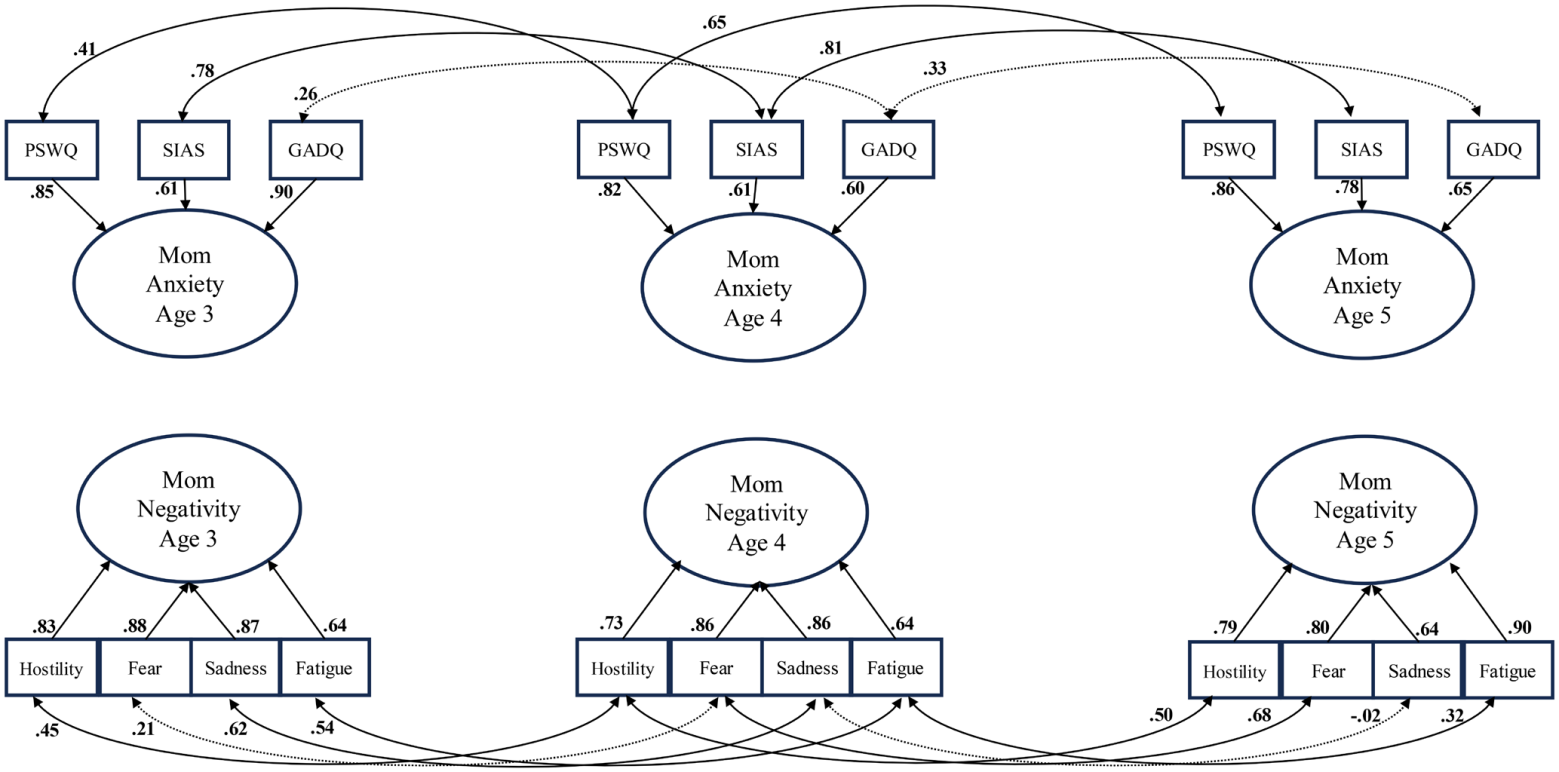
The measurement model. *N* = 119. All estimates are standardized estimates. Estimates represented by solid lines are significant at *p* < 0.05. Correlations from ages 3 to 5 were removed from figure to increase readability, but were estimated as follows: PSWQ *r* = 0.77, SIAS *r* = 0.71, GADQ *r* = 0.31 [n.s.], hostility *r* = 0.54, fear *r* = 0.11 [n.s.], sadness *r* = −0.17 [n.s.], fatigue *r* = 0.16 [n.s.]. Estimates added per modification indices were: GADQ age 4 correlations with SIAS age 5 *r* = 0.10 [n.s.] and PSWQ age 5 *r* = −0.07 [n.s.], sadness age 5 correlations with fatigue age 3 *r* = 0.56 and fatigue age 4 *r* = 0.62.

To confirm the structure of the latent variables, data were fit to a measurement model that included latent variables for maternal negativity and maternal anxiety but did not contain any autoregressive or crosslagged pathways (Figure 2). Correlations of the same measure over time were included in an effort to eliminate method variance from the latent factor. In addition, two correlations were added per model fit indices: the correlation of maternal GADQ scores at age 4 with both SIAS scores and PSWQ scores at age 5. Model fit was deemed to be adequate per a set of commonly used indices (RMSEA = 0.06 [0.05–0.08], CFI = 0.93, TLI = 0.90). As such, we proceeded with the test of the structural model.

### 2.3 Structural Model

**FIGURE 3:**
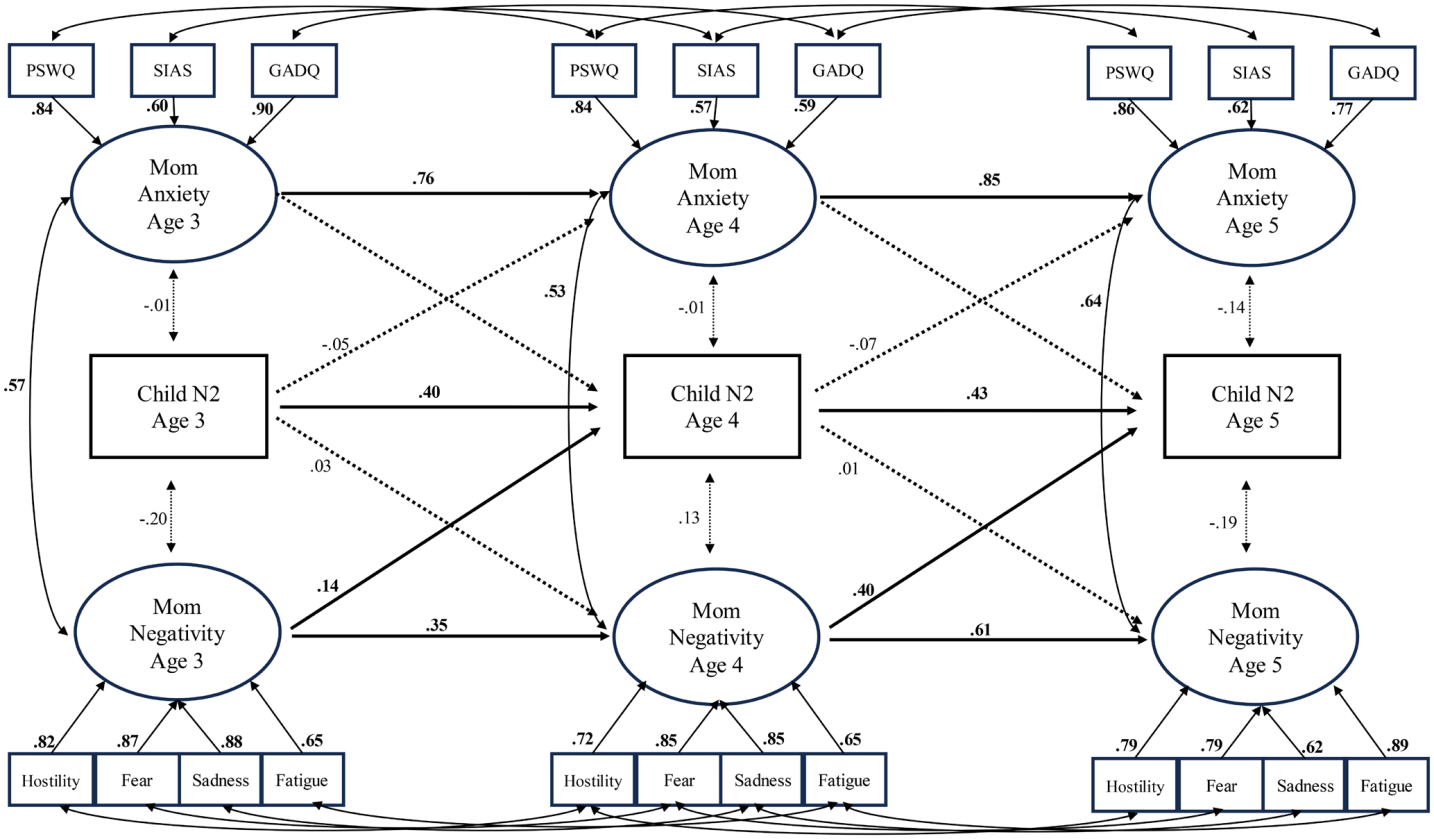
Results of a test of the structural model. *N* = 119. All estimates are standardized estimates. Bolded estimates are significant at *p* < 0.05. Dotted lines indicate nonsignificant paths. Correlations across manifest indicators were equivalent to the structural model. All residual variances were significant.

Fit was also deemed adequate for the structural model (Figure 3; RMSEA = 0.06 [0.05–0.08], CFI = 0.92, TLI = 0.90). Equality constraints in stability paths were added to test for the possibility of a more parsimonious model. Constraints were added only for autoregressive paths to allow for the possibility of increasing or decreasing crosslaged effects over time, which were of interest for primary hypotheses. Constraints on autoregressive paths were added such that constraints on the smallest raw difference (child N2) were added first and constraints on the largest raw difference (Mom Negativity) were added last. The addition of a constraint equating N2 amplitude stability across both temporal lags did not decrease model fit (Δ*χ* ^2^ = 0.52, df = 2) and so was included in the model. Similarly, neither an additional constraint on temporal lags in maternal anxiety (Δ*χ* ^2^ = 0.04, df = 1) nor a constraint on temporal lags in maternal negativity (Δ*χ* ^2^ = 2.57, df = 1) decreased overall model fit and so were retained in the model. All autoregressive pathways were significant, indicating that maternal anxiety, maternal negativity, and child N2 amplitudes were largely stable across assessments.

Cross‐sectional correlations between maternal characteristics and child N2 were all nonsignificant, suggesting that neither maternal anxiety nor negativity were contemporaneously associated with children's N2 amplitudes at any age. Similarly, children's N2 amplitudes did not significantly predict maternal characteristics at the subsequent assessment, suggesting an absence of child‐based effects on maternal anxiety and negativity. Maternal anxiety symptoms did not predict subsequent amplitudes for children's N2.

However, while greater maternal negativity at age 3 did not predict N2 at age 4, greater maternal negativity at age 4 predicted smaller N2 at age 5, suggesting a mother‐to‐child effect that emerged between ages 4 and 5. Supplemental analyses further suggested that effects were neither driven by a single form of anxiety symptoms nor simply attributable to overall propensities for emotional arousal (see Supporting Information).

## 3 Discussion

We offer new information about the developing N2 during the preschool years and its bidirectional associations with maternal emotion characteristics. In line with calls from the field for longitudinal work to advance understanding of the early development of childhood components of cognitive control, we tested longitudinal associations between N2, reflecting stability and change in a neural correlate of inhibitory control from ages 3 to 5, and its longitudinal associations with negative emotion and anxiety symptoms in mothers.

In contrast to other components that we have examined longitudinally, reflecting other facets of cognitive control (Brooker 2018), the N2 was largely stable during this period. Our use of residual scores follows recent revelations about the benefits of such an approach over subtraction‐based methods (Meyer et al. 2017), but precludes comparisons with existing work that would enable contrasts of N2 amplitudes between adults and children. However, stability in amplitudes of the N2 in our study was observed in a time window that largely overlapped with that used in adult studies with a similar task (e.g., Falkenstein et al. 1999; Harper et al. 2014). This similarity in the temporal window of the N2 for preschoolers and adults was somewhat surprising and suggests that the N2 may be earlier developing, or at least earlier stabilizing, than facets of cognitive control indexed by other ERPs, which show substantially later‐occurring time windows in children relative to adults (e.g., Brooker et al. 2019). An overlap in the timing of the N2 using similar tasks in adults and preschoolers is noteworthy, as heterotypic continuity in inhibitory control makes behavioral comparisons over time challenging, particularly beyond spans of more than 3 years (Petersen et al. 2016), and highlights a utility of the use of ERPs in developmental research.

Yet, conclusions about stability in N2 amplitudes do not preclude an influence for development. Our relatively broad temporal window for the N2, relative to work in older samples, is consistent with the possibility that improvements in inhibitory abilities in early childhood precede improvements in processing speed, which becomes more refined and efficient in later childhood (Best and Miller 2010). That is, processes of inhibitory control denoted by N2 may stabilize to an extent in early childhood but continue to become more efficient and less variable across development. Such a possibility is consistent with changes in N2 amplitude (Hoyniak 2017; Jonkman et al. 2007; Lewis et al. 2006) and in purported N2 source generators from childhood to adulthood (Jonkman et al. 2007) as well as with behavioral work on the development of inhibitory control. Moreover, in cross‐sectional work from our lab combining this sample with another sample of 3 year olds, we found broad variability in the number of preschoolers who demonstrated an individual‐level N2 effect (Mistry‐Patel et al. 2024), suggesting that patterns at the mean‐level belie individual differences in the development of the component. Indeed, inhibitory control shows the steepest growth during preschool years before beginning to level off during childhood (Geeraerts et al. 2021). Thus, similar to other physiological measures (Gunnar et al. 2009) future research will need to be thoughtfully planned and interpreted based on researchers' specific interests in mean‐level or individual‐level effects.

Though N2 amplitudes demonstrated considerable consistency over time, residuals in our model were notably larger than zero, allowing for some degree of change over time. Between ages 4 and 5, we found that at least a portion of the variance in the N2 that was unaccounted for by prior amplitudes was accounted for by levels of maternal negativity in the preceding year. This effect was fully absent for maternal anxiety symptoms, suggesting that domain‐specific negativity in mothers (i.e., anxiety symptoms) does not predict variability in preschoolers' N2 beyond what is accounted for by global levels of negative emotion.

There are two important caveats to the interpretation of results related to maternal anxiety symptoms in this sample. The first is that, although several mothers did meet thresholds for clinical levels of anxiety, our reliance on a community sample likely restricted the range of observed values of anxiety symptoms across participants, thereby limiting our ability to detect significant effects. Possible range restriction is most apparent in our measure of social anxiety, where scores were much more limited relative to the full possible range of the measure. In this way, our measure of maternal anxiety, targeted at clinical symptoms, assessed less normative and less varied experiences relative to our assessment of general negative affect. The second caveat is that maternal anxiety symptoms and negative emotion were allowed to covary in our model. As a result, effects of each construct reflect its independent contributions to children's subsequent N2, above and beyond shared variance across maternal characteristics. Thus, it is possible that maternal anxiety symptoms do predict characteristics of the N2, as has been demonstrated with older children (Tan et al. 2020); our results simply add nuance to such findings by offering longitudinal evidence that, similar to cross‐sectional work, this is unlikely to be an anxiety‐specific effect.

It is also worth noting that, although our model was grounded in a theoretical and empirical framework implicating a prominent role for anxiety in the development of children's inhibitory control, there may be utility in testing other discrete emotion pathways. For example, maternal depression appears to be uniquely linked to cognitive development in older children (Schäfer et al. 2023). Depression, with demonstrated and posited links to parental uninvolvement (Diego et al. 2006) is likely to operate through a different pathway than that outlined here (see Choe et al. 2023 for one possibility). This avenue will be a ripe area of exploration in future work.

Greater negative emotion in mothers predicted a smaller (less negative) N2 in offspring one year later. This pattern, whereby greater negative emotion predicts lower levels of inhibitory control in preschoolers, is consistent with both the scaffolding and imitation models of development. Namely, in the case of our inquiry, both models posit the same outcome; repeated interactions with dysregulated or overcontrolling parents, who may be less likely to either exemplify or scaffold successful control, likely impede the development of these skills in their children. At least one prior study with older children suggests that such an effect should be visible at the level of neural processing (Swingler et al. 2018). Our pattern of results extends prior findings by highlighting its visibility at the level of preschoolers' neural responses to events requiring the deployment of inhibitory control. Similar to the behavioral literature, it is difficult to know if such a process is visible even beyond the preschool years, given that our final assessment was at child age 5. Future work could be aimed at addressing this issue.

Unexpectedly, maternal negativity had associations with subsequent, but not contemporaneous, N2 in preschoolers. In fact, neither maternal negativity nor maternal anxiety symptoms were concurrently associated with preschoolers' N2 amplitudes. It is difficult to say precisely why this may be the case, but this pattern of findings perhaps offers a clue to differences in the temporal unfolding of the effects of maternal emotion on early neural activity relative to early behaviors. That is, it may be the case that while maternal anxiety levels may have more immediate impacts on children's behavior, visible as cross‐sectional associations, impacts at the level of neural functioning are shaped across a longer period of time. Indeed, these differences in the shape of developmental change across levels of inquiry are common and have served as an important point of consideration for researchers planning developmental studies. A second possibility is that maternal emotion characteristics, like negativity and anxiety, are concurrently related to N2 only when maternal anxiety manifests through low‐quality parenting behaviors (Geeraerts et al. 2021; Tan et al. 2020), though why this would not remain true over time is unclear. Indeed, it is difficult to find—in the literature—cross‐sectional correlations between maternal anxiety symptoms or maternal trait negativity and inhibitory control in preschoolers' that are not moderated by some maternal behavior, though this may reflect an overall tendency to publish more nuanced patterns of results.

Another possibility, consistent with our low bivariate correlations between maternal emotion characteristics and N2 amplitudes, is that maternal emotion does not specifically predict inhibitory control as called for by no‐go trials (as opposed to broader forms of cognitive control that would also be necessary during go trials, such as working memory or attention persistence). Given sufficient overlap, our use of residualized scores may “wash out” some of the variance that is most prominent in cross‐sectional associations. Indeed, one of the noted benefits of residual scores is that they minimize the possibility that effects are driven by the reference condition (here, go trials) rather than the condition of interest. As such, it will be valuable to amass a literature that adopts similar conceptual but different methodological approaches to the use of N2 to study inhibitory control in order to resolve this possibility.

A final note about the association between maternal negativity and subsequent N2 amplitudes in children is that this link appears to be strengthening over time; the association between age 4 maternal negativity and age 5 N2 was nearly three times greater than the same association between ages 3 and 4. To the extent that the N2 follows a developmental trajectory that overlaps with behavior (Geeraerts et al. 2021), this pattern of increasing effects may, in part, reflect the overall rapid maturation of inhibitory control during this period. A simultaneous decrease in error variance speculated above, as the timing of the N2 becomes less variable across individuals, would also contribute to an increasing effect size.

We were surprised to find no prospective effects of child N2 on maternal emotion characteristics during this period. Although the presence of child‐to‐caregiver effects is grounded in strong developmental theory (Bronfenbrenner and Morris 2007), effect sizes for such effects in our model were universally near zero. Other studies focused on the development of inhibitory control have similarly detected only mother‐to‐child effects (Geeraerts et al. 2021) despite broader evidence for child‐based effects on parent emotion (Brooker et al. 2015). One remarkable similarity across child‐to‐parent effects reported in this domain and at this age is that they largely comprise associations between children's *behaviors* and parent emotion characteristics rather than children's physiology. This is sensible given the nature of typical parent–child interactions: a parent's responses to the child will be largely driven by child behaviors, not by invisible (to parents) changes in physiology. To an extent, this possibility is similar to one raised regarding an absence of child‐to‐parent associations for symptoms of psychopathology. Specifically, child‐to‐parent effects appear to be most consistently detectable when the same rater reports on parent and child behaviors (Xerxa et al. 2021). Thus, there is clearly a role for shared method variance, but whether that shared variance should be considered error (due to the measure) or meaningful (in its elevation of the importance of parent perspectives) cannot be deciphered in the current work. Future studies will be needed to thoughtfully address these possibilities.

In addition to the role of observed behavior, consideration of the development of inhibitory control is warranted. Specifically, we believe it would be interesting to examine whether parents' perceptions of normative behavior at this age play a role. That is, given pervasive stereotypes in American culture that undercontrolled behaviors in children at this age are standard (e.g., “terrible twos,” “threenager”), might a parent simply be less emotionally responsive to undercontrolled behaviors if they view them as normative? If so, this low‐level responsiveness may appear in our data as an absence of child‐driven effects, as the child behaviors are consistent with parent expectations. Future work that explicitly assesses parent beliefs about the degree to which low levels of inhibitory control are normative at this age, or longitudinal studies that can track changes in child‐to‐parent effects as children are expected to become better controlled, will help to address this possibility and provide valuable insight into the developmental course of child‐to‐parent effects.

### 3.1 Limitations

Despite numerous strengths, this study is not without limitations. First, given somewhat modest effect sizes across child and parent effects, future work would benefit from a larger sample with the power to detect smaller effects. Second, given that there are cultural differences in parent perspectives about and the importance of developing high levels of inhibitory control, our results are largely not generalizable beyond the demographics of the sample from which they were derived. A more diverse sample, and particularly one that includes families from more traditional, subsistence‐based cultures in which self‐control is more rapidly developing and more explicitly valued (Li et al. 2018), will be necessary for understanding the generalizability of the current findings. Third, although the pattern of results fits our theoretical model that includes a role for parent behaviors, we did not directly assess parenting behaviors in the current study. As such, we are not able to directly test parent behaviors as a mechanism in an overall process and are accordingly limited in the degree to which we can delineate the trajectory by which parent characteristics may affect the development of cognitive control during the preschool years. Similarly, direct observations of parents' negative emotions, particularly if drawn from contexts that require children to exert cognitive control, may offer unique information and the processes by which parent behaviors and displays influence the development of cognitive control in young children. The incorporation of observed data will be an important direction for future work. The consideration of other maternal emotion characteristics, including maternal depression, that have demonstrated effects on early childhood neurodevelopment (Brooker et al. 2021; Kling et al. 2023) will also be critical for understanding the true nature of the process under study. Finally, we also note that while our use of N2 ERPs in children starting at age 3 is novel in critical ways, it remains probable that the children from whom we were *least likely* to obtain usable N2 data were those with the lowest levels of inhibitory control. Given this possible floor effect, we are wary about overinterpreting the absence of significant effects seen here. If we expect to see the most robust child‐to‐parent effects for children with the lowest levels of inhibitory control, then it will be necessary to sharpen our skills for collecting usable ERP data from more challenging participants.

## 4 Conclusions

In sum, this work offers novel insight into the relations between maternal emotion characteristics and preschoolers' inhibitory control, measured using the N2, between ages 3 and 5. We found evidence that the N2 is largely stable in preschoolers during this period, distinguishing it from other ERP metrics linked to cognitive control domains. We also found that N2 amplitudes were predicted by maternal negative emotion, but not maternal anxiety symptoms, in the previous year. We found no evidence for child‐to‐parent (child N2 to maternal negativity or anxiety) during this period. This study provides a foundation for future research that uses the N2 to index inhibitory control in young children and for longitudinal studies aimed at identifying the relations between maternal emotion and inhibitory control in children that change and develop over time.

## Supporting information


Appendix S1

## Data Availability

The data and syntax that support the conclusions of this manuscript are available through the Open Science Framework at https://osf.io/wavrp/?view_only=9210cace008c4bd1b19956bce1ed32be. The materials necessary to replicate this study are not publicly accessible. Analyses were not preregistered.
