# Supplementary material for: The N2 During Preschool: Temporal Stability and a Test of Bidirectional Effects With Maternal Emotion Characteristics in a White, European‐American Sample
Source: Child Dev. 2025 Jul 18;96(5):1825–38. doi: 10.1111/cdev.70004 (PMC12379866; doi:10.1111/cdev.70004)
Supplement: Supplementary file 1 — Appendix S1 [file CDEV-96-1825-s001.docx]

**Supplementary Information**

**Purpose**

This supplement contains additional details about the study method and analyses that are intended to clarify and contextualize the results of the manuscript.

**Method**

*Training and practice phases for the Go/No-Go task.* The training and practice phases of the Go/No-Go task comprised two parts. First, children were asked to respond to a series of laminated pictures of study stimuli by pushing the button (in response to asteroid pictures [go trials]) or demonstrating that they would “just wait” (in response to spaceship pictures [no-go trials]). Participants were required to get all 10 responses on the laminated photos correct to show that they understood what they needed to do.

Once participants demonstrated that they understood the task, they completed a set of computerized practice trials. Because children had already demonstrated an understanding of the task rules, there were not a minimum number of correct responses that children needed to make. The practice trials primarily served to get children acclimated to the speed of the task. Auditory feedback was provided to help learn this element of the task. On the rare occasion that children primarily gave incorrect responses on practice trials, they were allowed to repeat the practice trials.

**Results**

*Changes in maternal anxiety rates over time.* A sharp decline in maternal anxiety rates from Phase 1 and 2 prompted statistical tests for possible selective dropout, whereby mothers who were most anxious at Age 3 dropped out by age 4, resulting in artificially reduced rates of anxiety. Results from analyses ultimately suggest that this was not the case. Mothers with GADQ data at Age 4 did not differ in Age 3 scores from mothers without GADQ data at age 4 (*t*(118) = 1.48, *p* = 0.14). An inspection of the raw data suggests that that at age 4, a much larger than expected proportion of mothers responded to the questionnaire by saying that they simply do not experience high levels of worry, precluding the creation of a summed total of symptoms.

*Separating maternal social anxiety and generalized anxiety symptoms.* Anxiety scores reflecting social (SAD) and general (GAD) symptoms were composited because we had no *a priori* expectations that differences in dimensions of anxiety would lead to different effects on N2 development in children.

As a purely statistical check of whether findings were driven by or dependent on a specific domain of anxiety, we ran (a) a model including only the social anxiety measure (SIAS), and (b) a model excluding social anxiety (focused on worry and general anxiety). The model including only social anxiety (Figure 1; RMSEA = .08, CFI = .91, TLI = .88) showed a slightly worse fit than the original model (RMSEA = .06, CFI = .92, TLI = .90). This model returned a marginal effect of child N2 on maternal social anxiety from age 4 to age 5 (β = -.12, SE = .07, *p* = .08); this effect was not present, though also would not have been interpreted as significant, in the original model. The effect from maternal negativity to child N2 between child ages 4 and 5 remained significant (β = .28, SE = .11, *p* < .05), consistent with the original model.

In contrast, the model of general anxiety/worry, which excluded social anxiety (Figure 2; RMSEA = .05, CFI = .96, TLI = .95), largely replicated the model reported in the results of the manuscript; the significant path from maternal negativity to child N2 became non-significant at both age intervals (β_3-4_ = -.02, SE = .17, *p* = .93; β_4-5_ = .26, SE = .14, *p* = .07). This is likely related to a large correlation between maternal negativity and mothers’ levels of general anxiety, limiting the variance that could predict child outcomes in the partialed effect. This may suggest that individual differences in mothers’ social anxiety (that are independent from maternal negativity) are more likely to be affected by children’s levels of cognitive control, such that greater N2 in children is associated with more social anxiety in mothers at subsequent assessments.

*Figure 1: Rerunning model with only Maternal Social Anxiety*


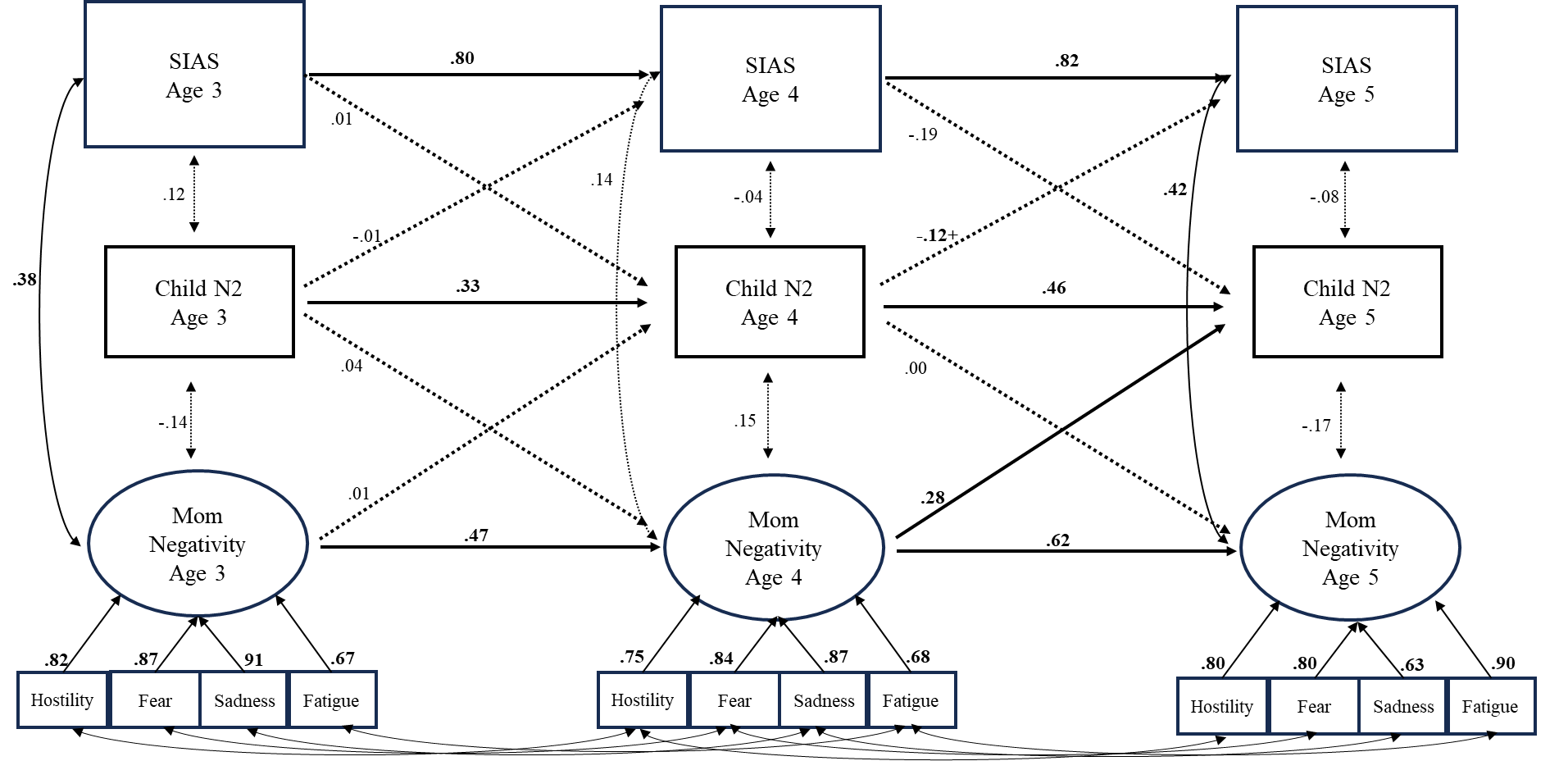


*Figure 2: Rerunning model with only symptoms of General Anxiety and Worry*


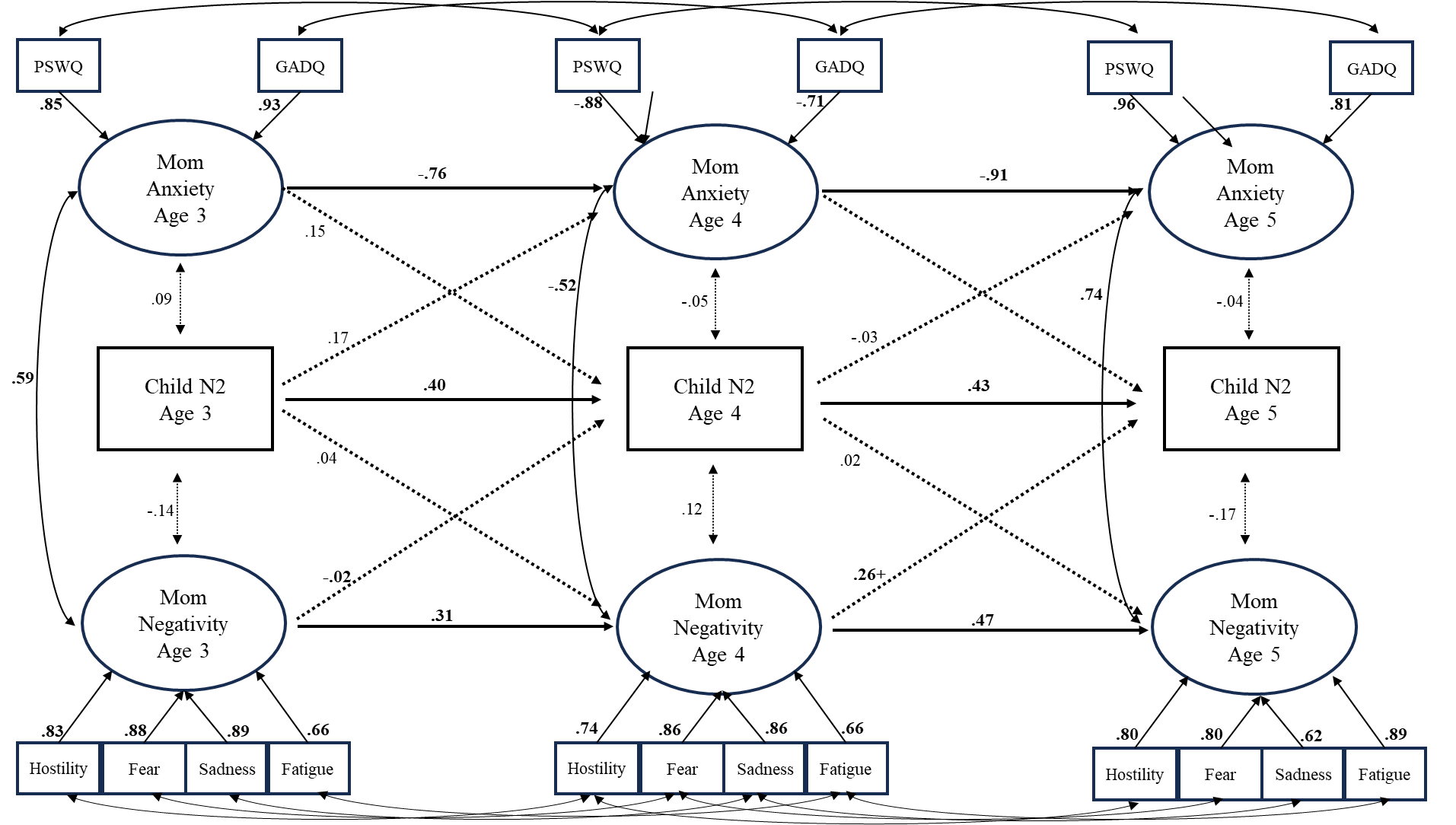


*Testing the specificity of effects to negative emotional arousal.* To test the specificity of effects to negative emotional arousal, and not more global emotional arousal, which would include positivity, we reran our statistical model using the positive items from the PANAS (i.e., maternal positivity) in place of the negative items from the PANAS (i.e., maternal negativity). This model (RMSEA = .05, CFI = .97, TLI = .96) returned no significant effects of child N2 on maternal positivity, either from age 3 to 4 (β = .01, SE = .11, *p* = .96) or from age 4 to 5 (β = .05, SE = .09, *p* = .57). Similarly, maternal positivity did not significantly predict child N2 from age 3 to 4 (β = .06, SE = .12, *p* = .63) or age 4 to 5 (β = -.04, SE = .11, *p* = .71).
